# Reflections of the quality of primary care in Canada and Israel

**DOI:** 10.1186/s13584-018-0243-y

**Published:** 2018-08-03

**Authors:** Richard H. Glazier

**Affiliations:** 1 Institute for Clinical Evaluative Sciences, Toronto, Canada; 2 Centre for Urban Health Solutions at St. Michael’s Hospital, Toronto, Canada; 3 Family and Community Medicine at St, Michael’s Hospital and University of Toronto, Toronto, Canada

## Commentary

Rachel Podell and her colleagues at the National Program for Quality Indicators in Community Healthcare in Israel (QICH) have provided a clear and engaging description of the quality of primary care provided to the elderly in Israel including changes over time, variation across sub-groups, and comparisons with other countries. Over a 13 year timeframe, most of the included process measures improved substantially with fairly minor differences between demographic groups and largely favourable comparisons with other countries. Other evidence from QICH indicates improvements in process measures for other age groups and conditions [1] and in enhanced equity and improved disease-specific outcomes including mortality [2]. Israel has one of the most advanced systems of monitoring the quality of primary care [3] and the OECD has noted the excellence of Israeli primary health care [4].

There are few direct comparisons of primary care available between Canada and Israel, but Canadian primary care compares relatively unfavourably with ten other developed countries in a number of measures including timely access to care, after-hours care, electronic medical record use and audit and feedback for quality improvement [5]. More concerning is that few of these measures have improved over a number of years despite a major policy focus on primary care [6] and investments in payment reforms and formation of groups and inter-professional teams [7]. While some areas such as cancer screening and diabetes care have improved [8], many aspects of primary care quality have been static over time and there are several examples of where reforms have not achieved their objectives [9, 10].

Differences in performance trajectories could relate to the major structural differences in primary care between Canada and Israel. While Canada has universal health insurance coverage for necessary physician and hospital services, most physicians practice privately, are paid mainly through fee-for-service and have few accountabilities to the health care system. Many Canadians lack a regular source of primary care [11] and there is little or no competition between primary care practices or groups as the majority are full and are not accepting new patients [12]. No Canadian province has completely implemented electronic medical records in primary care [5]. In contrast, Israel has the advantage of universal enrolment in one of four competing health maintenance organizations, employed physicians and other providers, full capture of electronic medical record encounters, prescriptions and tests and a robust quality reporting mechanism using population-based data.

It is unclear which of these mechanisms alone or in combination, and which additional factors have driven improvements in primary care processes and outcomes in Israel and it is therefore challenging to understand precisely what lessons Canada and other countries could learn from the Israeli example. Nonetheless, several key elements that are present in Israel and often missing in Canada are likely necessary for driving substantial improvements in population health outcomes. For example, many Canadian jurisdictions lack access to fundamental patient-level data such as prescribing, test results, blood pressure measurements, smoking status and body weights. In every jurisdiction a substantial proportion of the population lacks a primary care provider and in many jurisdictions those that have one are not registered or rostered with that provider, making attribution to a provider challenging. Electronic medical records are now used by most primary care physicians in Canada but many practices are unable to use their own data for quality improvement [5] and only a minority receive routine feedback on performance [13, 14]. In the absence of attributable data and mechanisms for accountability there are limited opportunities to use quality measurement as a strategy to improve population health. Canada also lacks the organizational, administrative and support structures of Israel’s health maintenance organizations [15].

Of course there are many contextual differences between countries that could drive differences in primary care quality and in changes over time. Canada has a vast land mass with 1/100th the population density as Israel, providing major challenges in care for remote communities. Canada has a higher proportion of seniors and higher net migration than Israel [16], both of which could increase need for care. Yet Canada spends 25% more per GDP on health than Israel and has a higher proportion of health spending in the public sector [17], suggesting that it receives lower value for its spending.

There are reasons for optimism for Canadian primary care. While accessing care can be challenging, once patients see a primary care provider they report very good patient experience and preventive care ratings are the highest of eleven countries [18]. Routine data extraction from electronic medical records is occurring for disease surveillance, research and quality improvement [14] and has the potential to be scaled up to close to the population level. Canada’s 14 health care jurisdictions mean that country-wide data and inter-jurisdictional comparisons are challenging but there is a commitment to build a National Data Platform to help surmount those barriers [19]. Group-based governance models are increasing in primary care, as is a move to alternate payment systems that could be accompanied by greater accountability and greater support for quality improvement.

## Conclusions

The Podell at et al. article provides a clear and compelling description of data-driven improvements in the quality of primary care provided to the elderly in Israel. Canada and other countries can learn from the advances in data, measurement, feedback, and organization of care that are now applied routinely to ongoing quality improvement in Israel, with impressive results. In contrast, little improvement in outcomes has taken place in Canadian primary care despite substantial policy advances and investments. The Israeli experience suggests that future developments designed to improve care and outcomes should include measurement infrastructure, formal reporting, accountability mechanisms, and management systems to address gaps and inequities in care.
